# Fiber-optic drug delivery strategy for synergistic cancer photothermal-chemotherapy

**DOI:** 10.1038/s41377-024-01586-z

**Published:** 2024-09-03

**Authors:** Yongkang Zhang, Jie Zheng, Fangzhou Jin, Jie Xiao, Ni Lan, Zhiyuan Xu, Xu Yue, Zesen Li, Chengzhi Li, Donglin Cao, Yifei Wang, Wenbin Zhong, Yang Ran, Bai-Ou Guan

**Affiliations:** 1 Guangdong Provincial Key Laboratory of Optical Fiber Sensing and Communications, Institute of Photonics Technology, Jinan University, Guangzhou, China; 2 College of Physics & Optoelectronic Engineering, Jinan University, Guangzhou, China; 3 The MOE Key Laboratory of Tumor Molecular Biology, Jinan University, Guangzhou, China; 4 Institute of Biomedicine, College of Life Science and Technology, Jinan University, Guangzhou, China; 5 Department of Laboratory Medicine, the Affiliated Guangdong Second Provincial General Hospital of Jinan University, Guangzhou, China; 6 Department of Interventional Radiology and Vascular Surgery, The First Affiliated Hospital of Jinan University, Jinan University, Guangzhou, China

**Keywords:** Optical sensors, Fibre optics and optical communications

## Abstract

Chemotherapy is one of the conventional treatments for cancer in clinical practice. However, poor delivery efficiency, systemic toxicity, and the lack of pharmacokinetic monitoring during treatment are the critical limitations of current chemotherapy. Herein, we reported a brand-new antitumor drug delivery strategy that harnesses an optical fiber endoscopically therapeutic probe. The fiber probe carries photosensitizers in the fiber core and antitumor agents on the fiber surface mediated by a temperature-responsive hydrogel film, giving rise to an activable photothermal-chemotherapy that orchestrates the localized hyperthermia and thermal-stimuli drug release to the tumor lesion. Furthermore, the dynamical drug release and in-situ temperature can be real-time supervised through the built-in fiber sensors, including the reflective Mach–Zehnder interferometer and fiber Bragg grating, to visualize the therapy process and thus improve the safety of treatment. Compared with conventional methods, the fiber-optic drug delivery can adequately take advantage of the chemotherapeutics through collaboratively recruiting the photoheating-mediated enhanced permeability and the hydrogel particle-assisted high drug retention, shedding new light on a “central-to-peripheral” drug pervasion and retention mechanism to destroy tumors completely. The fiber-optic chemotherapy strategy incorporates precise drug delivery, accurate controllability of drug release, high drug permeability and retention in tumor, low off-target rate, and real-time drug release and temperature feedback, performing a straightforward and precise photothermal-chemotherapy pathway. More than that, the proposed strategy holds tremendous promise to provide a revolutionized on-demand drug delivery platform for the highly efficient evaluation and screening of antitumor pharmaceuticals.

## Abstract (web summary)

Analogous to a jet-fighter, the drug-loaded fiber-optic therapeutics can attack tumor synergistically combing direct PTT and controlled-released chemo-drugs regardless the tissue barrier, and monitor therapeutic dosage at the lesion.

## Graphical abstract

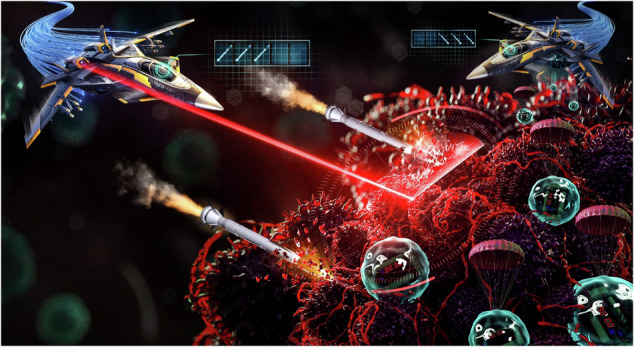

## Introduction

Cancer, caused by the pathophysiological alterations in cell division, is one of the most fatal diseases worldwide. More than 19.3 million new cancer cases and 10 million deaths were documented in 2020^1^. According to the report of American Association of Cancer Research (AACR), the new cases and deaths of cancer will reach 28 million and 16.2 million in 2030, respectively^2^. Tumorectomy, radiotherapy, and chemotherapy are the principal plans of the standard of care. Compared with the other counterparts, chemotherapy simply transports therapeutic agents to the tumor through intravenous injection or oral administration, featuring its advantages of non/minimal invasion and free of ionized radiation. In this regard, high accumulation of the drug into the tumor lesion is critical to improve the efficacy and safety of treatment^3^. However, the routinely used passive vascular or gastrointestinal delivery of chemotherapeutic drug often faces the challenge of low drug accumulation (only 1% of total dose of administration can arrive at tumor)^4,5^. More importantly, the substantial amount of dissociative drugs would inevitably damage the normal tissues and organs mediated by circulatory system, leading to serious side effects, such as cardiotoxicity, nephrotoxicity, hepatoxicity, or pulmonary fibrosis^6^. Therefore, poor delivery efficiency significantly impedes the effectiveness of drug therapeutics and even the accuracy of drug evaluation^7^.

Tumor-targeted-controlled drug release is a feasible way to improve delivery efficiency. It relies on the responsive nanocarriers to load and guide agents to tumors, and then unlock them under stimuli, such as pH, enzymes, redox, temperature, electromagnetic fields, light, and ultrasound^8,9^. Particularly, photoheating-mediated activation of drug is a promising scheme to elicit on-target releasing thanks to the excellent spatiotemporal controllability and non-invasiveness of laser radiation^10,11^. The photothermal effect results in immediate tumor debulking arisen from the localized hyperthermia, which can also improve the effectiveness of chemotherapeutic drugs through remodeling the tumor microenvironment (TME), such as blood flow, vascular permeability, extracellular pH, interstitial fluid pressure, facilitating the combination with chemotherapy for enhancing antitumor effect^12–15^. Nevertheless, the limited tissue penetration of external light radiation, time-consuming and insufficiency of drug retention, and lack of in-situ drug release monitoring during the treatment discourages the current photothermal-chemotherapy technology, no matter in drug research and clinical application^16–23^. Several drug delivery systems had utilized optical fiber to tackle the above-mentioned problems. However, the real-time sensing function and synergistic treatment are lacking^24–26^.

**Fig. 1:**
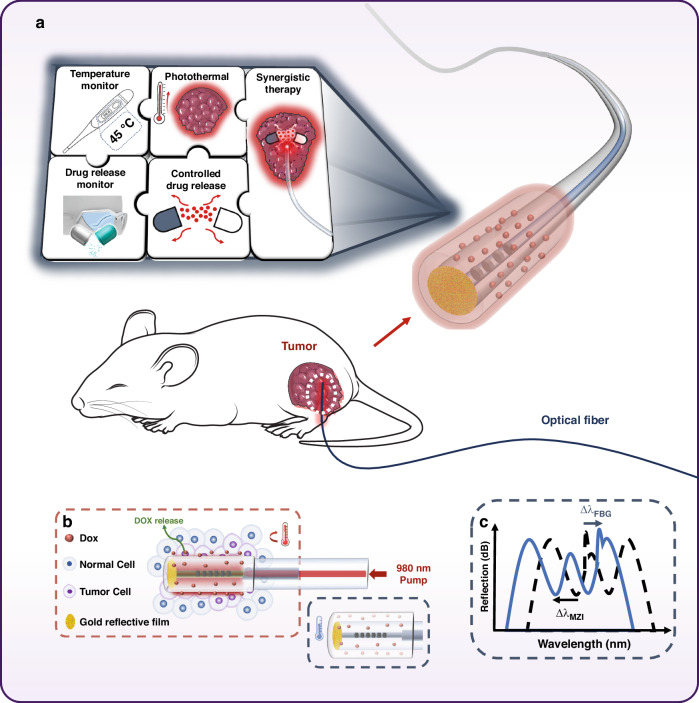
Schematic diagram of optic-fiber drug delivery system with the drug controlled-release and in-situ monitoring functions to implement the photothermo-chemotherapy. **a** The fiber-optic therapeutic probe features the minimal invasive nature to target the tumor in vivo. The fiber probe has photothermal therapy, drug delivery and thermally controlled release, temperature and drug release monitoring which can realize synergistic therapy as the pieces of puzzle displayed on the diagram. **b** The schematic diagram drug release of optic-fiber therapeutic probe with a Dox@Agarose film. The low-melting agarose carrying Dox are coated on fiber surface, which can be well-reserved under the melting threshold. In the operation, Dox will be precisely released from the agarose film at the tumor lesion as the localized temperature was increased by transmitting the 980 nm pump laser into the fiber probe. **c** Principle of the in-situ sensing of the fiber probe. The peak signal of FBG moving to longer wavelength indicates the increasing temperature, and interference signal of MZI shifting to shorter wavelength demonstrated the successful release of drugs. MZI Mach–Zehnder interferometer, FBG optical fiber Bragg grating, Δλ wavelength shift

Herein, we propose a versatile fiber-optic drug delivery and controlled-release system for realizing the high-efficacy photothermal-chemotherapy for cancer, as is shown in Fig. 1a. Besides shipping photons to deep tumor lesions, the optical fiber can load photothermo-sensitizers and chemotherapeutics, and precisely guide them targeting to tumor via interventional method^27–30^. As is depicted in Fig. 1b, once entering tumor, the fiber probe can be rapidly fueled by the incidence of laser to generate local hyperthermia, in which the fiber core dopants, i.e., the rare-earth ions, perform as photothermo-sensitizers. The localized high temperature then activates the low-melting-point agarose film, which is glued on fiber, to release the originally encapsulated doxorubicin (Dox). The abundant degree-of-freedom of optical fiber allows the layout of multiple integrated sensors, such as Mach–Zehnder interferometer (MZI) based on the reflective multimode-singlemode fiber splicing structure and fiber Bragg grating (FBG), to quantify the release of antitumor drugs and govern the temperature of lesion by transmitting data to clinicians in the operating room and even in remote area, as is illustrated in Fig. 1c. Compared with the conventional external laser radiation-based drug release method, the proposed fiber optic drug delivery not only overcomes the penetration limit for tackling deep and large tumors but also enhances the permeability and retention of antitumor drugs to scavenge the whole tumor based on the newly explored “central-to-peripheral” permeation mechanism. Specifically, minimal invasive fiber-optic photothermal therapy can enhance the vascular permeability of tumor and regulates the TME, rendering the drugs fully pervading throughout the tumor. Furthermore, the melted agarose hydrogel particles that wrap drugs can dock at the tumor with a longer period and continue to make use of Dox to destroy tumor cells, which share similar effect with transcatheter arterial chemoembolization (TACE) but with a more preferable drug-releasing rate. Either in vitro and in vivo experimental results confirm the pronounced efficacy of the fiber-optic drug delivery probe. The HepG2 tumors of xenograft can be completed eradicated through just one treatment with a 10-minute duration using just one optical fiber probe. The employment of agarose materials and Dox, which were both approved by Food and Drug Administration (FDA), and the photothermo-sensitizers, which are caged in the fiber core and taken out with the fiber after treatment, adequately guarantee the bio-safety of this new approach^31^. The results of drug biodistribution after treatment further discloses high tumor-targeted efficiency of the proposed drug delivery strategy, with no evidence of affecting normal tissues and organs. Those advantages confer this technique to lift beyond a therapeutic probe by providing a high-efficiency and compatible drug screening and evaluation strategy for the research and development of new anticancer drugs, and even the re-assessment of the drugs that had been ruled-out due to the systemic toxicity^32^.

## Result

### The characterization of sensing performance

**Fig. 2:**
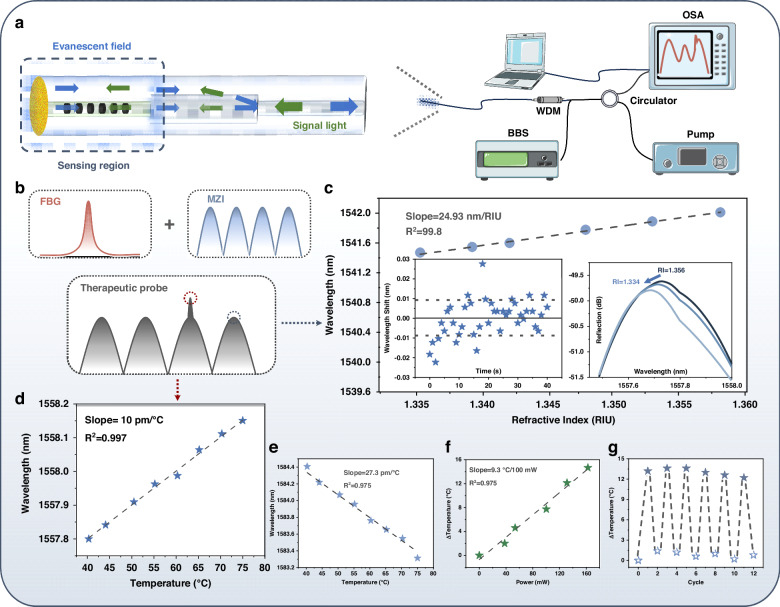
The characterization of sensing performance. **a** Schematic of optic-fiber therapeutic probe sensing. OSA optical spectrum analyzer, BBS broadband light source with a 600–1700 nm spectral range, Pump 980 nm laser pump, WDM wavelength division multiplexing of 980 nm and 1550 nm. **b** The Schematic of MZI, FBG, and Therapeutic probe’s spectrum. **c** Response of the optic-fiber probe to the change of ambient RI. Insert: (Left) long-term wavelength stability of the MZI immersed in the DI water at the room temperature. (Right) The spectrum of MZI recorded in different RI. **d** Temperature sensitivity of the built-in FBG. **e** Temperature response of MZI. **f** Response of optic-fiber probe with the increment of pump power. **g** The pump “on-off” cycle test for the optic-fiber probe (the power of pump is set to 160 mW for the “on” state). RI refractive index

Figure 2a shows the optical structure of the fiber drug delivery system. The sensing functions were realized by the in-line MZI and built-in FBG and the spectrum were showed in Figs. 2b and S2. According to the light path, the incident light (green) passed through the multimode fiber and then split into two propagation modes, i.e., the fundamental and higher order modes, respectively, as it enters the rare earth-doped single-mode fiber. Transmitting in the cladding of fiber, the higher order mode light held a larger evanescent field to interact with ambient. Reflected by the gold film coated on the fiber end-face, the light passing by different paths would re-encounter at the fusing point. The change of the optical path of higher mode light represented the variation of surrounding refractive index, enabling the measurement on the concentration of drug in the agarose film.

The RI sensitivity of MZI is crucial to monitor the Dox release. In our experiment, the RI sensitivity of MZI is 24.93 nm/RIU, as shows in Fig. 2c. Subsequently, the RI resolution of MZI and the long-term stability test of MZI the was also explored in Fig. 2c inset (left). The standard deviation of the wavelength shift is 9.5 pm, and the limit resolution is 1.1 × 10^−3^ RIU calculated by Eq. S1. This demonstrates its capability of converting a tiny alteration of RI to a distinguishable signal change and its potential for drug release sensing.

For the PTT treatment, the temperature is essential for drug release and photon-induced hyperthermia. Unfortunately, the MZI is also sensitive to temperature. The temperature cross-sensitivity of the sensing scheme significantly deteriorates the accuracy of the drug monitoring. Therefore, precise temperature monitoring is as well necessary and important. To address this issue, we inscribed a FBG directly in the rare earth-doped fiber for enabling an absolute temperature sensor.

The temperature sensitivity of the fiber optic structure was calibrated by a water bath. In Fig. 2d, e, the temperature sensitivity is −27.3 pm/°C (*R*^2^ = 0.975) and 10 pm/°C (*R*^2^ = 0.997) for the MZI and FBG, respectively. In the treatment, the temperature could be demodulated firstly by the FBG sensing. The temperature-mediated wavelength shift of MZI could be compensated by acquiring the real time temperature logged from the FBG and endowed with the system the accurate RI value, which represented the drug releasing. After that, the photothermal effect of the fiber system was evaluated by elucidating the relationship between the power of the 980 nm pump and the increasing temperature, which is discussed in Fig. 2f. The heating efficiency is 9.3 °C/100 mW(*R*^2^ = 0.99), and the near-linear curve indicates the potential of PTT in Fig. 2g.

### Characterization of drug release monitoring

**Fig. 3:**
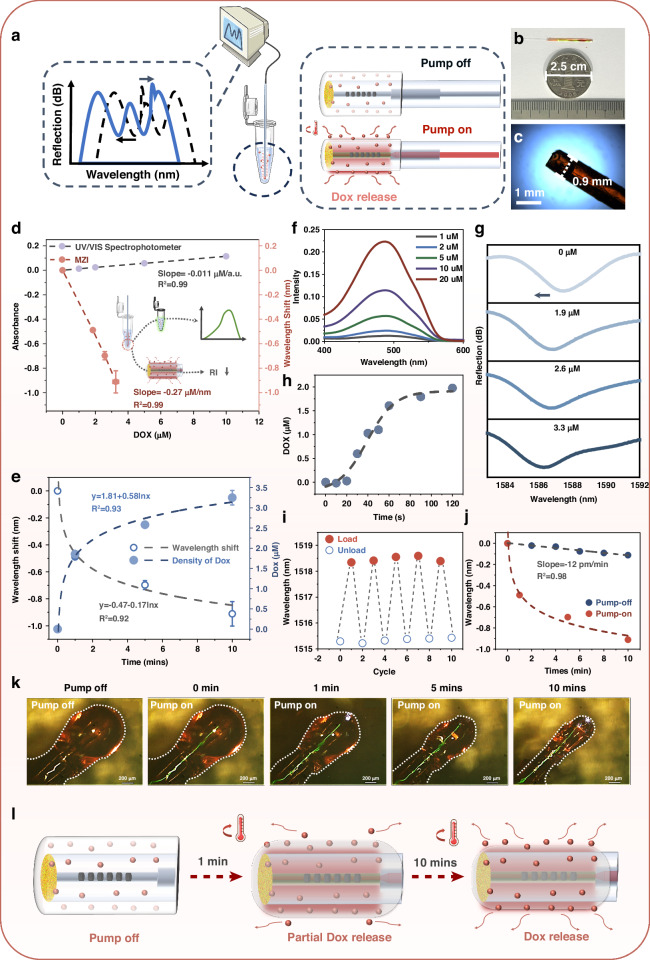
Characterization of drug release monitoring. **a** Schematic of Dox release sensing by optic-fiber therapeutic probe. **b** The photo of fiber-optic drug delivery system with Dox@Agarose comparing with a CNY 1 coin. The length of Dox@Agarose is ~ 2 cm. **c** The image of optic-fiber therapeutic probe under the microscope. The diameter of optic-fiber therapeutic probe is ~ 900 μm. **d** The concentration calibration of Dox in the aqueous solution through UV/VIS Spectrophotometer and the MZI. **e** The dynamic wavelength response of MZI to the Dox releasing through photothermal actuation. The Dox concentration was time-dependently calibrated by the UV-VIS spectrum. **f** Absorption spectra of Dox at different concentrations with the peak absorption at 500 nm. **g** Spectral evolution of MZI in dox releasing. **h** Dox releasing dynamic in the first 120 seconds. **i** Cycling test of drug releasing by coating Dox@Agarose on the fiber repeatedly. **j** The wavelength shift comparison between pump-on and pump-off. **k** The observation of drug release under the microscope. **l** The schematic of Dox release process

The chemotherapy agent release and sensing were then characterized, as is shown in Fig. 3a. Low melting point agarose is chosen owing to its photothermal controllable melting nature and excellent biocompatibility. The agarose possesses a phase transition mediated responsiveness to the change of temperature. At higher temperature, such as 65 °C, the hydrogel presents a molten state, which facilitates the mixing with the chemotherapy agent and decoration on the fiber surface via gluing. As temperature decreases to room temperature (approximately 25 °C), the liquid-like agarose will be solidified to perform as drug capsule, which forms a functional film (Length: ~ 2 cm, diameter: ~0.9 mm) on the fiber as is shown in Figs. 3b, c and S3. The coin-sized therapeutic probe with the coagulated agarose film, which encapsulates and thus strengthens the mechanical performance of the silica fiber device, would benefit for the following in vivo experiment.

Intriguingly, the hydrogel starts softening again as the temperature reaches a considerably low temperature of 40 °C and enables Dox release, as shown in Videos S1 and Videos S2^33^. In addition, 48 °C is the threshold between low-temperature photothermal therapy (LTPTT) and conventional PTT^34,35^. LTPTT is a more moderate therapy way to kill tumor cells without damaging the surrounding normal tissues. In this work, the temperature was controlled to 48 °C throughout the photothermo-chemotherapy treatment.

The Dox concentration calibration was studied first. A series of solutions with Dox concentrations ranging from 0 μM to 20 μM were prepared by mixing DI water with Dox in different ratios. The optic fiber therapeutic probe with Dox@agarose was prepared as described in the “Materials and methods” section. It is worth noting that drug loading can be controlled by adjusting the concentration of Dox and the diameter and length of the coating. The coat assembles on the fiber surface through a hollow cylinder mold made of polytetrafluoroethylene, which can be designed by a mold. Then, as the 980 nm laser was turned on with a power of 250 mW, the fiber probe was self-heated to 48 °C in a short time. Dox@agarose film was softened and then partly melted under heating, resulting in the releasing of Dox originally encapsulated in the agarose hydrogel. In Fig. 3d, f, the concentration of Dox is calibrated through the ultraviolet-visible absorption spectrum and MZI. The slope of sensing curve obtained from UV-VIS absorption and MZI were 0.011 a.u./μM (black line) and −0.27 nm/μM (red line), respectively.

Subsequently, the Dox release in the sensor is discovered. The fiber-optic probe with Dox@Agarose was immersed in 1.5 mL DI water at 37 °C, and then pumped by the 980 nm laser with the power of 250 mW. The Dox concentration in water in accordance with the pump duration was measured by recording the absorbance of solution using UV/VIS spectrophotometer. Correspondingly, the spectrum variation of MZI was also monitored. As is shown in Fig. 3g, a dip signal chosen from the interference spectrum moved to shorter wavelength as the drug releasing, which was resulted in the heat-induced melting of agarose film and Dox releasing away from fiber. In Fig. 3e, the black curve shows the evolution of the wavelength shift of MZI with regard to the duration of photoheating, which follows the log-linear relationship, *y* = −0.47-0.17ln x. The blue shifting of the dip signal could be transduced into decreasing of the refractive index of hydrogel film, indicating that the drug concentration of the film was lowering. The sensor gram of MZI agreed well with the spectral absorbance of the solution, although the two tendencies exhibited a mirror-like relationship. The absorbance-duration curve fits the log function, *y* = 1.81 + 0.58ln x, denoting that Dox particles were released into the solution from the hydrogel film. Both curves proved that the MZI sensor could monitor the Dox release during the tumor therapy. It is worth noting that the release speed is not the same in the duration, and it is evident that the release speed is higher at the first few minutes after pump actuation. Then, the Dox release speed is further explored for the first two minutes, as is shown in Fig. 3h. We can see that the release rate reaches plateau within 60 s, which guides us to set the proper photothermal actuation duration of one minute in the in vitro experiments. In the following, the Dox@Agarose film was repeatedly coated on one fiber probe to test the repeatability and reliability of drug releasing, as is shown in Fig. 3i. The wavelength shift >3 nm in each cycle indicates the consistency of the coating and drug releasing of the optic-fiber therapeutic probe. Finally, the stability of Dox@Agarose film in the normal condition is revealed in Fig. 3j. The slow rate of drug leakage (0.045 μM/mins) and the significant gap of wavelength shift of MZI between the “with pump” and “no pump” tests proves that the Dox leakage of film is negligible without pump injection, facilitating storage and transportation of the fiber probe for the practical use.

### In Vitro anticancer performance of the fiber optic drug delivery probe

**Fig. 4:**
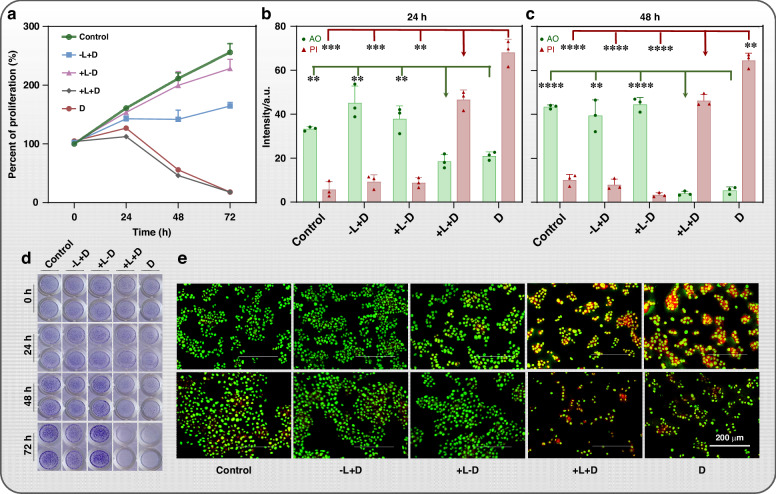
In vitro anticancer performance of the fiber optic drug delivery probe. **a** The proliferation curve of HepG2 cells in different groups; (**b**) The intensity and statistical distinctions of AO/PI staining of HepG2 cells after 24 h; and (**c**) 48 h treatments. (AO/PI: acridine orange/propidium iodide; live cells, green fluorescence; dead cells, red fluorescence). Statistical analysis is performed by Student’s *t* test (*t*-test). *p < 0.05; **p < 0.01; ***p < 0.001; ****p < 0.001. **d** The photograph of crystal violet staining after 0, 24, 48, and 72 hours in different treatments. **e** The fluorescence microscopy of AO/PI staining (live cells, green fluorescence; dead cells, red fluorescence) contained HepG2 cells after treatments. The scale bars are 200 μm

In vitro therapeutic effect of the fiber optic drug delivery probe was subsequently investigated. Five groups were set in the cell experiment as Table S1 shows: It’s worth noting that the label of +L means the probe is pumped with 980 nm pump and +D means the probe loads and releases the Dox. Group 1, no operation (blank control); Group 2, fiber with Dox@Agarose without 980 nm pump(-L+Dox); Group 3, fiber coated by Agarose without encapsulating Dox to perform photothermal only with 980 nm pump(+L-Dox); Group 4, photothermal actuation (with 980 nm pump) with Dox@Agarose (+L+Dox); Group 5, Dox only (Dox, 2 μM) without pump. The fiber probe with Dox@Agarose film was pumped in cell experiments lasting for 60 seconds in group 3 and 4. The HepG2 cells were cultivated, and the proportions of proliferation were calculated by Cell Counting Kit-8 (CCK-8), as is shown in Fig. 4a. The proliferation of +L+D group decreases to 18% and the statistical differences among Blank (256%) and +L-D (228%) groups at 72 hours in Fig. S5 proves the inhibition to cancer cell proliferation via successful Dox release. The crystal violet test (Living cells take up the dye and become stained) also confirmed this effect, as shown in Fig. 4d. The lighter or even transparent purple of the +L+D and D group shows the ability to eliminate cancer cells.

The AO/PI (acridine orange/propidium iodide; live cells, green fluorescence; dead cells, red fluorescence) was used to distinguish the live/dead cells, as Fig. 4e shows. The fluorescence intensity of AO/PI of +L+D group are 18.5 a.u./46.6 a.u. (AO/PI) after 24 h, and 33.3 a.u./5.7 a.u. (AO/PI) in Fig. 4b, c. Comparing with the Blank (24 h: 33.3 a.u./5.7 a.u.; 48 h: 43.4 a.u./10.1 a.u.), the lower intensity of AO and higher intensity of PI in +L+D group suggested the lethal toxic effect for HepG2 cells, which is consistent with the CCK-8 test results.

### In vivo tumor eradication

**Fig. 5:**
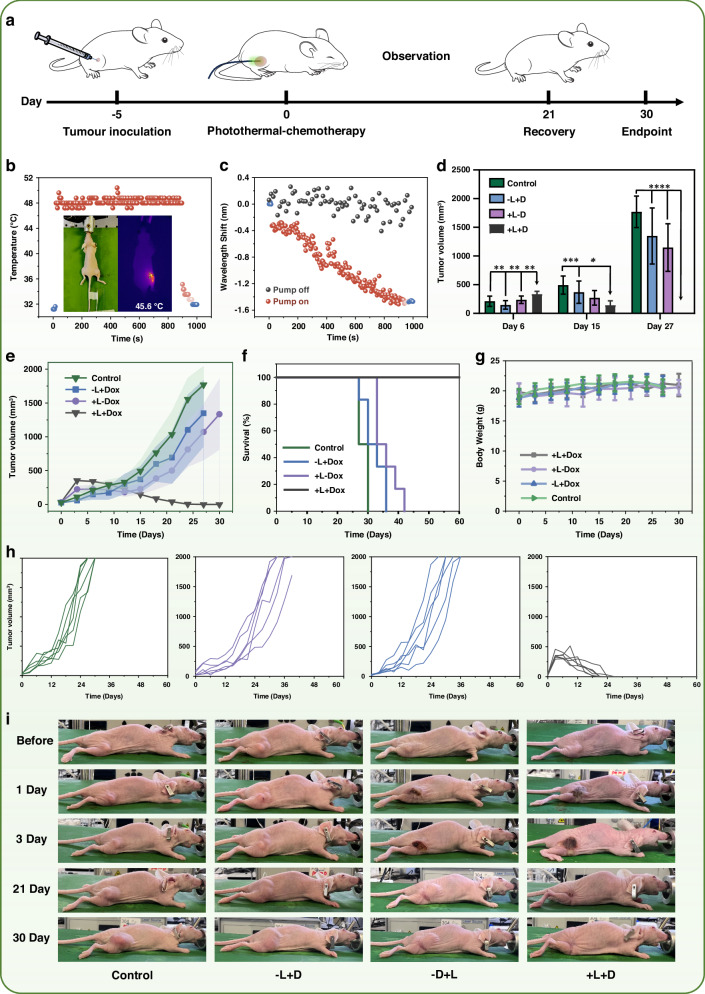
In vivo tumor eradication. **a** Schematic illustration and timeline of the photothermo-chemotherapy using the optic-fiber therapeutic probe with Dox@Agarose. **b** Temperature monitoring by FBG. Insert: The real treatment image on the left and the thermal image of mice bearing tumors during treatment on right. **c** The monitoring of MZI wavelength shift during the treatment. **d** Statistical distinctions among the groups at several typical time ends. Statistical analysis is performed by Student’s *t* test (*t* test). *p < 0.05; **p < 0.01; ***p < 0.001; ****p < 0.001. **e** Corresponding growth curves of tumors in different groups of mice at a measured time; *V*_0_ means the volume before treatment. The volume (mm^3^) = 1/2 × (tumor length) × (tumor width)^2^ (*n* = 6). **f** Kaplan-Meier survival curve of mice after different treatments. **g** Body weight of nude mice recorded every 3 days after treatment. **h** The tumor growth curves of each mouse in different groups. **i** Representative photographs of mice bearing in different groups sets before and after various treatments

We carried out animal experiments to test the potential of the optic-fiber drug delivery system with Dox@Agarose for in vivo application. The procedures for the in vivo tumor eradication experiment are shown in Fig. 5a and Videos S3. The tumor-bearing nude mice were prepared before treatment. The photothermal effect of the device was first studied. After the spearing of the sensor into the tumor, the temperature was monitored by a thermal camera and FBG during 10 mins of treatment, and the ∆T was about 45.6 °C in the NIR image and 48 °C in FBG in Fig. 5b. The discrepancy in temperature results is due to the differences in sensing depth. The FBG can accurately detect the photothermal effect of intratumor, but the thermal camera can only monitor the surface temperature.

Meanwhile, the MZI monitored the Dox release, as shown in Fig. 5c. The abrupt wavelength shift at the start of the pump proves the rising temperature of the sensor, and a 1.2 nm blue shift indicates that an amount of 4.4 μM of Dox was released during the treatment. Compared with the “pump-off” curve, only a 0.4 nm wavelength jitter is observed, which was resulted in from the mice breathing. This result validates the reliability of controlled drug release of the proposed optical fiber probe in vivo.

Based on the in vivo therapeutic effects, we conducted an in vivo antitumor study to validate the enhanced cancer therapy. Group 1, without operation (blank control); group 2, with Dox@Agarose only (-L+Dox); group 3, with photothermal therapy only( + L-Dox); group 4, with photothermal therapy and Dox@Agarose (+L+Dox) and group 5 with injection of Dox solution (+Dox). The tumor volumes were logged every three days. In line with the results of the tumor growth curve showing in Figs. 5e and S6a, the average tumor volume of +L+D group reached the maximum (353 mm^3^), and the lesions presented eschars within 3 days and finally vanished (0 mm^3^) in about 24 days, which showed statistical significance of difference to the other three groups in Fig. 5d. The cure rate reached 100% in the treatment group. The results demonstrated that the photothermo-chemotherapy strategy possessed an excellent tumor treatment effect. It is also worth noting that the average tumor volume was 1333 mm^3^ at Day 30 for +L-Dox group, while reached 2000 mm^3^ for the Blank and -L+Dox groups both at Day 27, inferring that the fiber PTT could inhibit tumor growth but with a limited outcome due to its insufficient heating range. The mice were sacrificed as the tumors reach 2000 mm^3^ tumor, and thus a Kaplan-Meier survival curve was obtained, as shown in Fig. 5f. The +L+D group kept alive for 60 days without evident recurrence, much longer than all the other groups (Blank control: 30 days; -L+Dox: 36 days; +L-Dox: 42 days). In addition, the average body weight of mice in +L+D group were maintained at ~19 g, which showed no significant difference regarding the other groups, as is shown in Figs. 5g and S6b. Body weight loss is one of significant side effects of chemotherapy^36^. However, in our experiment, the steadily increasing weight curve revealed that no acute side effects were induced by the proposed photothermal-chemotherapy. The monitoring of tumor volume of the mice in different groups after treatment were portrayed in Fig. 5h, which agree well with the photographs of mice presented in Fig. 5i. The eschars appeared at tumor lesions after treatment, leading to false rises of average tumor volumes in the +L+D, +L-D, and +Dox group. In +L+D group, the lesions were recovered within 21 days, which was longer than +L-D group due to the continuous antitumor effect mediated by slow Dox release. As a consequence, the tumors in +L+D group were completely removed.

### Mechanism of drug permeate and retention

**Fig. 6:**
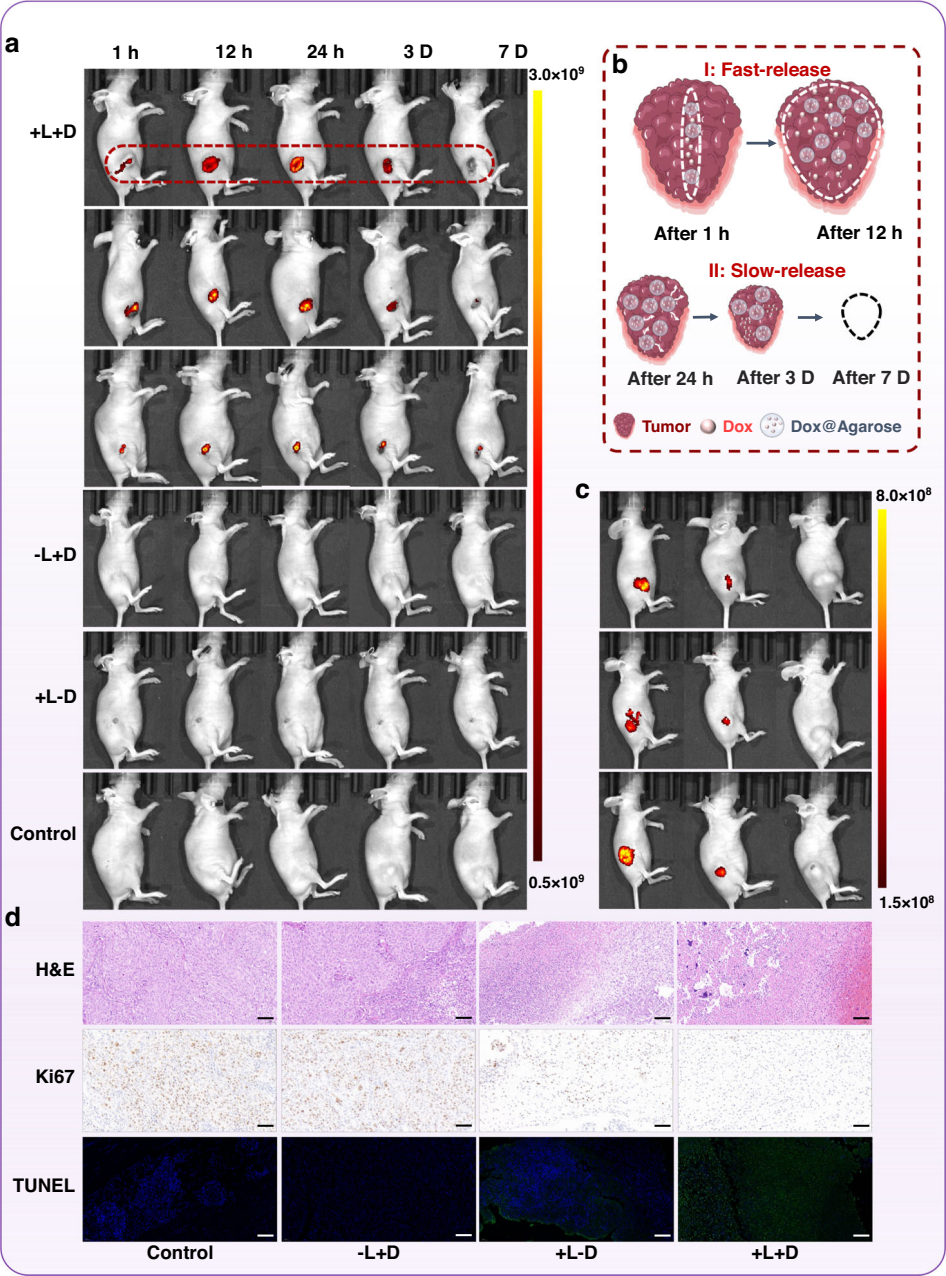
Dox diffusion and therapeutic mechanism in mice after different treatments. **a** Fluorescence images of mice at 1 h, 12 h, 24 h, 3 d, 7 d after the photothermo-chemotherapy based on optic-fiber drug delivery system (h, hour, d, day). **b** Schematic illustration of Dox diffusion and therapeutic mechanism after photothermo-chemotherapy based on the optic-fiber probe. **c** Fluorescence images of Dox@Agarose degradation in tumor without photothermal actuation. **d** H&E, Ki67 immunohistochemistry and the TUNEL staining of tumor sections after 24 h in different treatments. Scale bars of H&E, Ki67 are 200 μm, and the scale bars of TUNEL are 100 μm

The excellent therapeutic results from +L+D group intrigue us to further explore the synergistic therapeutic mechanism of the treatment. Taking advantage of the autofluorescence nature of Dox, the evolution of Dox in the mouse after the treatment can be visualized by the in vivo optical imaging system (IVIS). The in vivo fluorescent images were recorded at 1 h, 12 h, 24 h, 3 days, and 7 days after the treatments of different schemes. The dynamic change of fluorescence of Dox is presented in Fig. 6a. We can see the fluorescence mapping in the +L+D group only. Surprisingly, -L+D group did not show any fluorescence because the Dox@Agarose film was completely moved out of the tumor with the retrieval of the fiber probe without the laser activation. The phenomenon also confirms the robustness and reliability of the designed fiber probe. The results elucidate that both laser-induced photothermal actuation and Dox@Agarose loading were featured for the synergistic therapy, which are in accordance with the results of in vitro experiments. It is worth noting that the fluorescent patterns formed straight lines at 1 h throughout the group of +L+D, which indicates that the drug release is in the vicinity of the optical fiber in the tumor at an early stage. Subsequently, the fluorescent signal expanded to the complete tumor within 12 hours, featuring a “central-to-peripheral” permeability mechanism. The fluorescent pattern showed the peak intensity at 1 day after the treatment. Then the fluorescence gradually reduced but could last for a week, which is much longer than the direct injection of Dox into the lesion, as is shown in Fig. S7. In this regard, the mechanism of the synergistic therapy can be deduced, as is shown in the Fig. 6b and Videos S4. As the pump laser was actuated, a part of Dox dose was rapidly released outside the optical fiber due to the melt of the agarose, allowing the dissociative Dox to attack tumor cells. The residual Dox was still encapsulated in the agarose film but which was tailored into small agarose gel particles due to the heating. Here, the photothermal treatment performed as not only a trigger to the release of drug and segmentation of agarose hydrogel, but also as a stimulus to expand the vascular of tumor and reconfigure the TME, conferring a significant boost to the drug permeability. The small agarose gel particles greatly improved the retention of the Dox, which allows for the continuous TACE-like anticancer effects resulted in from the biodegradation of agarose particles^37^. This “central-to-peripheral” photothermo-chemo therapeutic strategy can scavenge the malignant cells and TME throughout the tumor, showing a different characteristic in contrast with the conventional phototherapy that utilizes the laser radiation from outside.

The biodegradation of Dox@Agarose is also discussed in Fig. 6c. A bulk Dox@Agarose gel with the same volume to the agarose film on the optical fiber was directly injected into the tumor. The fluorescence of Dox lasted for 30 days, which is much longer than +L+D group because the laser-induced photoheating could rapidly release a portion of Dox and tailor the residual Dox@Agarose into smaller and more degradable pieces. More importantly, the Dox release through the merely passive biodegradation showed no obvious effect on tumor suppression even in 30 days. It means that the ultra-slow rate of passive drug release cannot overwhelmingly inhibit the rapid growth of tumor. Therefore, by orchestrating those mechanisms, the fiber-optic drug delivery strategy holds an optimal drug retention and release rate, giving it an edge over the other routinely used drug delivery methods, such as passive drug release method and direct DOX administration without encapsulation^38^.

The therapeutic effects were validated by the histological report, as are illustrated in Fig. 6d. After 24 h of treatment, three mice of each group were randomly selected for histological and immunohistochemical analyses. The hematoxylin and eosin (H&E, show the morphologic structure of various tissue or cellular components) of tumor tissues had hemorrhage and necrosis in +L-D group, and these effects were enhanced in a substantial degree for +L+D group. Immunohistochemical assays of Ki67 (antigens associated with proliferating cells, more colored dots represent high expression of cell proliferation) and TUNEL (apoptotic cells fluorescing in green) revealed a reduction in the number of proliferative tumor cells and an increase in the number of apoptotic cells, validating the excellent therapeutic outcome of the +L+D group.

The fluorescence images are shown in Fig. S8, and the H&E staining images (to show tissue cell morphology: the nucleus appears purplish-blue, and the cytoplasm and extracellular matrix appear red in color) of nontargeted organs (hearts, livers, spleens, lungs, kidneys) are shown in Fig. S9. The lack of differences among the four groups demonstrated that photothermal chemotherapy had negligible effects on normal organs and tissues. The +L+D treatment also shows no significant differences to the Blank control on the normal organs using CD31 (for assessment of angiogenesis, abnormalities occur when organs are damaged) and Caspase-3 immunostaining (for assessment of apoptosis, abnormalities occur when organs are damaged) were not significantly different between the +L+D treatment group and the Blank control group, as shown in Figs. S11 and S12. Those results confirm the outstanding biosafety of the proposed strategy, which relies on the high efficiency of drug delivery and retention in the tumor rather than normal organs. More than that, the high efficiency of on target drug transportation can either facilitate the full utilization of therapeutic agents for strongly supporting the investigation and assessment of new drugs regardless of the issues, such as solvent adaptability, toxicity due to the circulation, and rapid metabolic rate.

### Demonstration of mini-invasiveness and versatility of the fiber probe

**Fig. 7:**
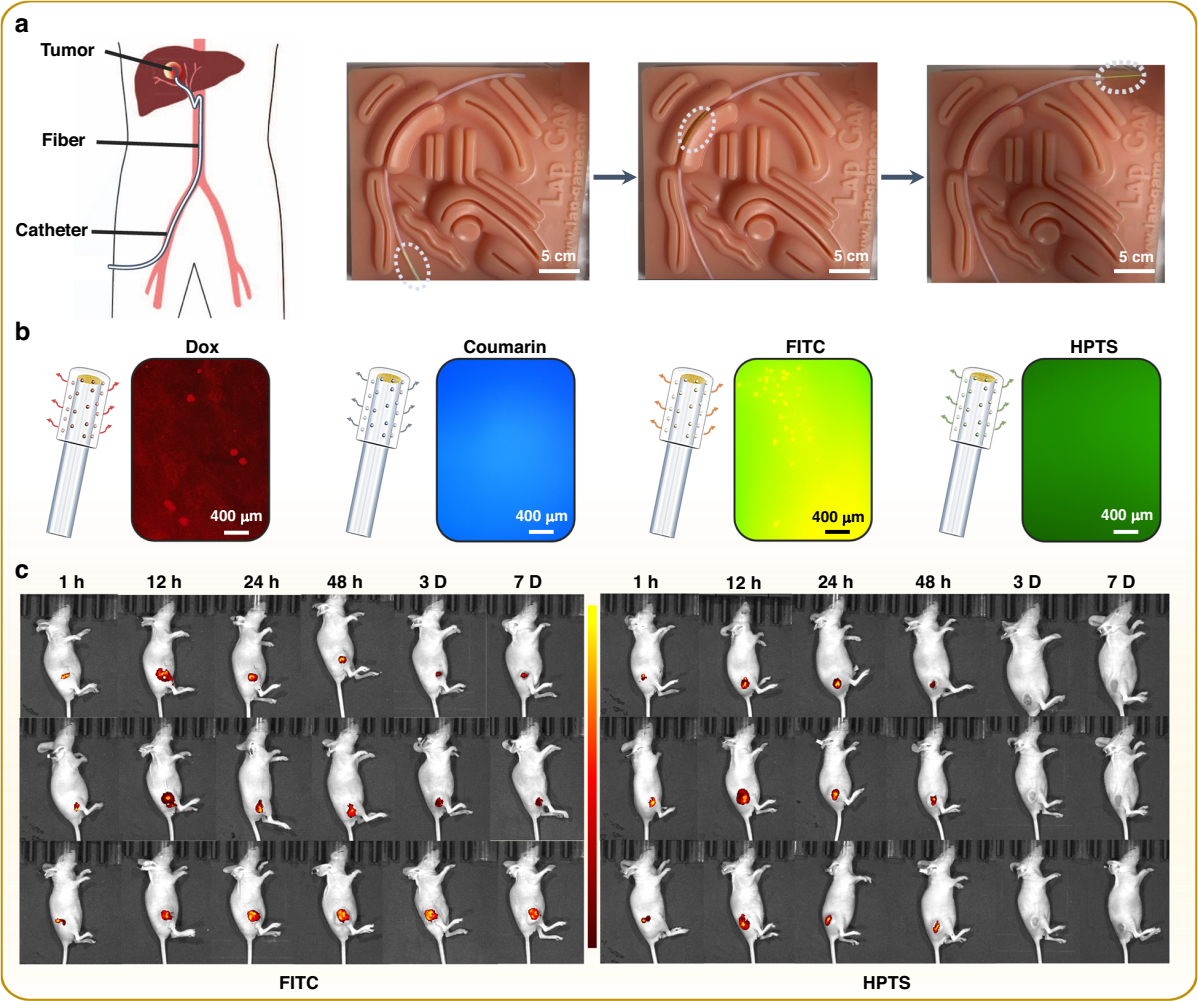
A mini-invasive drug delivery platform. **a** Schematic diagram and photographs of the fiber-optic targeted drug releasing compatible with interventional therapy. **b** The in vitro release (Dox, Coumarin, FITC, HPTS) under fluorescence microscope. Coumarin (325/450 nm) and HPTS (454/511 nm) are soluble agents, resulting in the uniform patterns in color due to the homogeneous spreading after releasing. Dox (485/585 nm) is slightly soluble, leading to small particles observed in the picture. FITC (495/530 nm) is insoluble, giving rise to largest particles. **c** Fluorescence images of mice at 1 h, 12 h, 24 h, 3 d, 7 d after the drug releasing using optic-fiber drug delivery system (h, hour, d, day) using FITC (fluorescein isothiocyanate) and HPTS (8-Hydroxypyrene-1,3,6-trisulfonic acid, trisodium salt)

The interventional treatment is a low grade of invasiveness that can achieve the focus by interventional devices (intervention catheter or trocar)^39^. Interventions are minimally invasive due to the small size of the interventional device and can reach most lesions through body channels, for example, the blood vessels. The optic-fiber drug delivery probe is natively compatible with the interventional treatment due to the compact size and flexible structure. As are demonstrated in Fig. 7a and Videos S5, using a phantom of abdominal cavity, the fiber probe can reach the deep target by sliding into the blood vascular model (the inner diameter of vascular is ~ 1.5 mm) and conduct targeted drug release. It is also suitable for interventional therapy at different curvatures, as is shown in Fig. S12.

In fact, more than 90% of total drug candidates fail to be approved for clinical trials due to systemic delivery-initiated unmanageable toxicity and drug characteristics, such as molecular size, dissolvability, tumor-targeting ability, and metabolism, in different animal models^32^. Nevertheless, our optic-fiber therapeutic probe is able to avoid the above problems, indicating that most drug candidates can be re-administered through this fiber-optic drug delivery system. Therefore, the versatility of the fiber delivery probe between different drugs should be tested. Then, we replaced Dox with several fluorescent agents, Coumarin, FITC and HPTS, with distinct solubility and emission performances. The observation of the in vitro release of different agents under fluorescence microscope are shown in Fig. 7b and their in-vivo distribution under IVIS system are shown in Fig. 7c. Even water-insoluble dye (FITC) is successfully released. Most importantly, the mechanisms of “central-to-peripheral” and short-time TACE are well-confirmed by the in vivo imaging, regardless the solubility and emission characteristics of the agents, strongly encouraging us to envisage a versatile drug screening and evaluation platform based on the fiber-optic drug delivery.

## Discussion

In summary, we propose an attractive fiber-optic drug delivery strategy and demonstrate a high-efficiency photothermal-chemotherapy platform for cancer treatment to overcome the limitations mentioned above. The optical fiber loading photothermo-sensitizers (rare-earth dopants) in the core and chemotherapeutics on the surface can reach the lesions precisely through the minimal invasive method regardless of the depth of tumor location. In order to achieve a safe and monitorable treatment, the sensing modules of MZI and FBG are integrated in the optic-fiber probe, which can monitor the release of antitumor drugs and govern the temperature of lesions respectively. Furthermore, in vitro and in vivo experiments both demonstrated the pronounced efficacy of the fiber-optic drug delivery probe. The biodistribution of Dox using IVIS imaging and histopathologic examination after treatment adequately confirms the high tumor-targeted efficiency and drug permeability of the proposed drug delivery strategy, and the circumvention of the drug-induced adverse effect on normal tissues and organs.

**Fig. 8:**
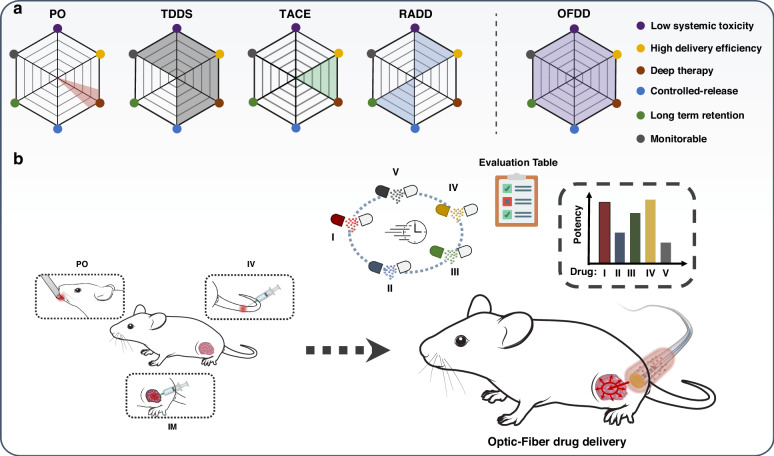
The prospect of fiber-optic drug delivery. **a** The Radar chart for the comparison among the category of non/minimal invasive drug delivery strategies, such as PO^40^, TDDS^41^, TACE^42^, RADD^43^, and OFDD. **b** Schematic diagram of revolutionized on-demand drug delivery platform based on optic-fiber drug delivery for the highly efficient evaluation and screening of antitumor pharmaceuticals. PO Per os, oral administration, TDDS targeting drug delivery system, TACE transcatheter arterial chemoembolization, RADD radiation-assisted drug delivery, OFDD optic-fiber drug delivery, IV intravenous injection, IM intramuscular injection;

More than a direct anticancer scheme, photothermal therapy can increase the vascular permeability of the tumor and regulate the tumor microenvironment to boost the uptake of the cancerous cells to the drug throughout the tumor and thus enhance the efficiency of tumor scavenging. Furthermore, rather than the conventional photoheating based therapy that utilizes external laser radiation, which compromises the utilization of laser power and completeness of tumor eradication, the proposed drug delivery harnesses a fiber-optic mediating intra-lesion heating pathway. This interior photothermal effect yields a newly explored “central-to-peripheral” drug pervasion mechanism to further enhance the permeability and retention of Dox to scavenge the complete tumor. Compared with the frequently-used drug sending method, such as intravenous and intratumor injection, and oral administration, the fiber-optic drug delivery strategy can greatly enhance the intratumor drug concentration, permeation, and retention without being subjected to drug complexity, solubility and organ toxicity, as is shown in Fig. 8a and Table S2. The proposed fiber-optic drug delivery strategy promises a high-performance synergistic therapy to fight against cancer. The above-mentioned advantages allow this technique beyond a simple therapeutic probe by performing as a high-efficiency, versatile drug screening and evaluation platform, which gathers the high targeting delivery rate, accurately activable drug release, enhanced permeability and retention, and dose and temperature monitoring, for the research and development of new anticancer drugs, as is expected in Fig. 8b.

Future studies will focus on: (1) adding the cancer diagnostic in the fiber probe; (2) improving the sensitivity of sensor and increasing the parameters of sensing that can further visualize the condition of tumor in the treatment. More innovative concepts such as TFBG would be applied in this strategy in the ensuing studies; (3) conducting tests on more kinds of anticancer drugs in different animal models to make a broad comparison for building an evaluation standard; (4) taking advantage of the anti-electromagnetic and compact nature of optical fiber device to push forward the technique from laboratory to clinic medicine. In this regard, the fiber optic drug delivery probe is be naturally compatible with the current diagnostic and mini-invasive therapeutic techniques. For example, the magnetic resonance and ultrasound imaging can provide navigation and assist drug releasing monitoring for the fiber probe; the fiber probe can also be aided by the interventional surgery to punch above its weight through the guidance of blood vessel intervention catheter and percutaneous intervention stylet.

For a broader perspective, in the pharmaceutical field, only less than 10% of total drug candidates would be approved after clinical trials. It is worth noting that a substantial part of failed drug candidates is due to the systemic delivery-initiated unmanageable toxicity^32^. Besides, the discrepancies of drug characteristics, such as molecular size, dissolvability, tumor-targeted ability, and metabolism with regard to different animal models may increase the difficulty in objective evaluation of the drug efficacy. Those hurdles severely hinder the steps of current research and development of new anticancer drug. What if the delivery efficiency, systemic toxicity and pharmacokinetic management could be significantly improved by guaranteeing precise drug targeting to the lesion without circulating the entire body? A large number of dead drugs may be recalled and drug investigators will obtain a good dose of courage to embrace new chemical techniques or even AI design for drug development through optic-fiber drug delivery.

## Materials and methods

### Fabrication of Mach–Zehnder interferometer with FBG

The MZI used reflective multimode, single-mode and multimode (MSM) fusing structure, and the single-mode fiber was replaced with active fiber, as Fig. S1 shows. After MZI fabrication, the Bragg grating was inscribed in the active fiber through the phase mask method. A 193 nm UV excimer laser (Compex 110, Coherent, Inc.) and a phase mask with a 1072.5 nm pitch (IBSEN photonics, Denmark) were used for the inscription. The laser had a frequency of 50 Hz. A cylindrical lens converged the beam to enhance the energy density to 300 mJ/cm^2^ per pulse. In the process of inscribing, an optical spectrum analyzer (OSA) with a spectrum range of 600-1700 nm and an ultra-wideband light source (Golight) with a range of 1250-1650 nm were needed to demodulate and record the optical signals in this process. The resolution of OSA was set to 0.05 nm to log the tiny spectral variation. The inscription time was about 10 minutes. The gold film was coated on the MZI reflective end by an ion spectrometer (SBC-12, KYKY). Finally, an MZI with FBG was obtained, named optic-fiber probe.

### Fabrication of Dox@Agarose

Low melting point agarose (Macklin, Shanghai, China) and Dox (Aladdin, Shanghai, China) powder prepared to fabricate Dox@Agarose. Low melting point agarose was dissolved in Deionized water at 60 °C and cooled to a semi-liquid state, and then the weighed Dox powder was added to the agarose solution (the concentration of Dox in the mixed solution was 800 μg/mL). After that, the above mixture was rapidly cooled to room temperature (26 °C) to obtain Dox@Agarose.

### Fabrication of the optic-fiber therapeutic probe with Dox@Agarose

A 2 cm long section of polytetrafluoroethylene cannula (1.2 mm inner diameter) was prepared, and it was placed over the active fiber. Then, Dox@Agarose in the mixed state (heated at 60 °C) was drawn by a 1 ml syringe and injected the mixture into the polymer cannula. After waiting for the Dox@Agarose cooled to room temperature, the polymer cannula was removed, and the active fiber loaded with Dox@Agarose was prepared, named “Optic-fiber therapeutic probe with Dox@Agarose.”

### Characterization of refractive index sensitivity of MZI

A series of solutions with different refractive indexes ranging from 1.33 to 1.36 were prepared by mixing DI water with ethanol in different ratios. The refractive index of each solution was calibrated through a hand-held refractometer (PAL-RI, ATAGO, Japan) at a temperature of around 25 °C. Subsequently, the MZI was immersed in each solution, and the spectra were recorded.

### Characterization of refractive index resolution of MZI

The wavelength jittering was interrogated by immersing the device in DI water for 40 min and monitoring the wavelength drift of the reflected reference peak.

### Characterization of materials

The morphology of the Optic-fiber therapeutic probe with Dox@Agarose’s surface was measured by field-emission scanning electron microscopy (SEM, Apreo 2 SEM). Ultraviolet-visible near infrared spectrophotometer observed UV–vis absorbance spectra of samples (SHIMADZU, UV-3600 plus, Japan).

### Cell lines and cell culture

The details are in Supplementary Information.

### CCK-8 assay

The details are in Supplementary Information.

### AO/PI staining

The details are in Supplementary Information.

### The preparation of in vivo tumor eradication

Isoflurane (RWD Life Science Co. Ltd., China) was guided into tumor-bearing mice using a small animal anesthesia machine (R580S, RWD Life Science Co. Ltd., China). The flow rate of isoflurane was 2 L min^-1^, and the density was 1.5%. The 980 nm laser with power of 250 mW was transported through the fiber. The tumor’s real-time thermal dates were obtained simultaneously using the photothermal imager and optical fiber temperature sensor (FBG) and quantified by BM_IR and MATLAB software. IR imaging during the photothermal test was acquired by the photothermal imager (FOTRIC 225 s, FOTRIC, China) to reference the fiber photothermal effect.

### Animals

The four to five-weeks-old female BALB/c-Nu mice [BALB/cJGpt-Foxn1nu/Gpt] were purchased from Charles River (Foshan, China). Animal experiments were approved by Jinan University’s Institute of Experimental Animal Ethics Committee (Approval number: IACUC-20230506-05), and all mice were kept in Laboratory Animal Center, Jinan University. Nude mice were maintained under specific pathogen-free (SPF) conditions for one week before the study.

### Establishment of cancer xenografts

Human liver cancer cell line HepG2 (5 × 10^6^ cells) suspended in PBS (200 μL) was inoculated into the subcutaneous right leg of BALB/c-Nu mice (six weeks old, female). When the tumor size reached 50–80 mm^3^, tumor-bearing mice were randomly divided into 4 groups (*n* = 6): group 1, without operation (blank control); group 2, with Dox@Agarose only (-L+D), fiber loaded Dox@Agarose was impaled into tumors and waited for 10–15 minutes; group 3, with photothermal therapy only (+L-D), only active fiber under 250 mW 980 nm pump was placed into tumors and waited for 10–15 min; group 4, with photothermal therapy and Dox@Agarose (+L+D), active fiber loaded Dox@Agarose under 250 mW 980 nm pump was placed into tumors and waited for 10-15 min. After 24 h post-treatment, two tumors in each group were randomly selected, weighed, photographed, and stained with H&E, Ki67, TUNEL in tumor tissues. A series of organs (heart, liver, spleen, lung, kidney) from two nude mice in each group were randomly selected, weighed, photographed, and stained with H&E, cleaved-caspase 3, CD31. After different treatments, the nude mice were fed continuously for 30 days, and the volume of the tumors as well as the weight of the nude mice were recorded. The mice’s body weight and tumor volume were measured and recorded every three days. The tumor volume was calculated according to the following equation: V = a× b^2^/2 (a was the tumor length and b was the tumor width).

### Biodistribution analysis

IVIS (In vivo optical imaging system) Spectrum (PerkinElmer) was used to take the in vivo images of mice because the Dox. The 1 h, 12 h, 24 h, 3 days, and 7 days after the treatments in vivo were measured, respectively. The biodegradability of Dox@Agarose was studied by the fluorescence of Dox in the IVIS spectrum. The organs (heart, liver, spleen, lung, and kidney) and tumors from the blank control and +L+D groups were taken to analyze the drug diffusion in Fig. S8. A 520 nm wavelength light was used as the excitation source, and 570 nm was detected as the emitted light.

### Histological and immunohistochemical analyses

The details are in Supplementary Information.

### Test of the versatile drug screening and evaluation platform

Low melting point agarose (Macklin, Shanghai, China) or Dox (Aladdin, Shanghai, China) or Coumarin (Macklin, Shanghai, China) or FITC (Dogesce, Beijing, China) or HPTS (Aladdin, Shanghai, China) powder prepared to fabricate Drug@Agarose. Low melting point agarose was dissolved in saline at 60 °C and cooled to a semi-liquid state, and then the weighed drug powder was added to the agarose solution (the concentration of drug in the mixed solution was 800 μg/mL). After that, the above mixture was rapidly cooled to room temperature (26 °C) to obtain Dox@Agarose.

### Statistical analysis

All experiments were performed more than three times individually. Statistics are expressed as mean ± standard error of the mean (SEM). All statistics were processed using GraphPad Prism software (GraphPad Software, La Jolla, USA). Differences among the three groups were assessed using Student’s *t* test (*t*-test). *P < 0.05 was considered a statistically significant difference.

## Supplementary information

Supplemental material for Fiber-optic drug delivery strategy for synergistic cancer photothermal-chemotherapy

The observation of Dox@Agarose melting by photothermal under the microscope

The observation of drug release by optic-fiber therapeutic probe under the endoscopy

The treatment process of photothermo-chemotherapy based on the fiber-optic drug delivery system

The animation of drug release

The combination of optic-fiber therapeutic probe and interventional therapy

## References

[1] Chhikara, B. S. & Parang, K. Global cancer statistics 2022: the trends projection analysis. *Chem. Biol. Lett.* **10**, 451 (2023).

[2] Williams, P. A., Zaidi, S. K. & Sengupta, R. AACR cancer progress report 2022: decoding cancer complexity, integrating science, and transforming patient outcomes. *Clin. Cancer Res.* **28**, 4178–4179 (2022). 36129416 10.1158/1078-0432.CCR-22-2588

[3] Bhatia, S. N. et al. Cancer nanomedicine. *Nat. Rev. Cancer* **22**, 550–556 (2022). 35941223 10.1038/s41568-022-00496-9 PMC9358926

[4] Jain, R. K. & Stylianopoulos, T. Delivering nanomedicine to solid tumors. *Nat. Rev. Clin. Oncol.* **7**, 653–664 (2010). 20838415 10.1038/nrclinonc.2010.139 PMC3065247

[5] Kim, S. M., Faix, P. H. & Schnitzer, J. E. Overcoming key biological barriers to cancer drug delivery and efficacy. *J. Control. Release* **267**, 15–30 (2017). 28917530 10.1016/j.jconrel.2017.09.016 PMC8756776

[6] Cavaletti, G. Peripheral neurotoxicity of platinum-based chemotherapy. *Nat. Rev. Cancer* **8**, 72–72 (2008). 10.1038/nrc2167-c1 18159632

[7] Li, Z., Zou, J. H. & Chen, X. Y. In response to precision medicine: current subcellular targeting strategies for cancer therapy. *Adv. Mater.* **35**, 2209529 (2023). 10.1002/adma.202209529 36445169

[8] Senapati, S. et al. Controlled drug delivery vehicles for cancer treatment and their performance. *Signal Transduct. Target. Ther.* **3**, 7 (2018). 29560283 10.1038/s41392-017-0004-3 PMC5854578

[9] Wang, Y. F. & Kohane, D. S. External triggering and triggered targeting strategies for drug delivery. *Nat. Rev. Mater.* **2**, 17020 (2017). 10.1038/natrevmats.2017.20

[10] Li, X. S. et al. Clinical development and potential of photothermal and photodynamic therapies for cancer. *Nat. Rev. Clin. Oncol.* **17**, 657–674 (2020). 32699309 10.1038/s41571-020-0410-2

[11] Liu, Y. J. et al. Photothermal therapy and photoacoustic imaging via nanotheranostics in fighting cancer. *Chem. Soc. Rev.* **48**, 2053–2108 (2019). 30259015 10.1039/C8CS00618K PMC6437026

[12] van der Meel, R. et al. Smart cancer nanomedicine. *Nat. Nanotechnol.* **14**, 1007–1017 (2019). 31695150 10.1038/s41565-019-0567-y PMC7227032

[13] Dunne, M., Regenold, M. & Allen, C. Hyperthermia can alter tumor physiology and improve chemo- and radio-therapy efficacy. *Adv. Drug Deliv. Rev.* **163-164**, 98–124 (2020). 32681862 10.1016/j.addr.2020.07.007

[14] Phung, D. C. et al. Combined hyperthermia and chemotherapy as a synergistic anticancer treatment. *J. Pharm. Investig.* **49**, 519–526 (2019). 10.1007/s40005-019-00431-5

[15] Ribeiro, T. P. et al. Nanomaterials in cancer: reviewing the combination of hyperthermia and triggered chemotherapy. *J. Control. Release* **347**, 89–103 (2022). 35513211 10.1016/j.jconrel.2022.04.045

[16] Cheng, D. B. et al. Intracellular self-immolative polyprodrug with near-infrared light guided accumulation and in vivo visualization of drug release. *Adv. Mater.* **34**, 2109528 (2022). 10.1002/adma.202109528 34933400

[17] Chen, W. et al. Smart chemical engineering-based lightweight and miniaturized attachable systems for advanced drug delivery and diagnostics. *Adv. Mater.* **34**, 2106701 (2022). 10.1002/adma.202106701 34643302

[18] Wang, R. et al. In vivo gastrointestinal drug-release monitoring through second near-infrared window fluorescent bioimaging with orally delivered microcarriers. *Nat. Commun.* **8**, 14702 (2017). 28281530 10.1038/ncomms14702 PMC5353702

[19] Pang, Q. et al. Smart flexible electronics-integrated wound dressing for real-time monitoring and on-demand treatment of infected wounds. *Adv. Sci.* **7**, 1902673 (2020). 10.1002/advs.201902673 PMC7080536 32195091

[20] Yong, T. Y. et al. Tumor exosome-based nanoparticles are efficient drug carriers for chemotherapy. *Nat. Commun.* **10**, 3838 (2019). 31444335 10.1038/s41467-019-11718-4 PMC6707218

[21] Yang, K. K. et al. Supramolecular polymerization-induced nanoassemblies for self-augmented cascade chemotherapy and chemodynamic therapy of tumor. *Angew. Chem. Int. Ed.* **60**, 17570–17578 (2021). 10.1002/anie.202103721 34041833

[22] Tan, A. R. et al. Fixed-dose combination of pertuzumab and trastuzumab for subcutaneous injection plus chemotherapy in HER2-positive early breast cancer (FeDeriCa): a randomised, open-label, multicentre, non-inferiority, phase 3 study. *Lancet Oncol.* **22**, 85–97 (2021). 33357420 10.1016/S1470-2045(20)30536-2

[23] Zhao, P. F. et al. NIR-driven smart theranostic nanomedicine for on-demand drug release and synergistic antitumour therapy. *Sci. Rep.* **5**, 14258 (2015). 26400780 10.1038/srep14258 PMC4585834

[24] Chin, A. L. et al. Implantable optical fibers for immunotherapeutics delivery and tumor impedance measurement. *Nat. Commun.* **12**, 5138 (2021). 34446702 10.1038/s41467-021-25391-z PMC8390758

[25] Ding, Y. P. et al. Electrospun fibrous architectures for drug delivery, tissue engineering and cancer therapy. *Adv. Funct. Mater.* **29**, 1802852 (2019). 10.1002/adfm.201802852

[26] Talebian, S. et al. Biopolymers for antitumor implantable drug delivery systems: recent advances and future outlook. *Adv. Mater.* **30**, 1706665 (2018). 10.1002/adma.201706665 29756237

[27] Zhu, Y. J. et al. Rational design of biomaterials to potentiate cancer thermal therapy. *Chem. Rev.* **123**, 7326–7378 (2023). 36912061 10.1021/acs.chemrev.2c00822

[28] Mehrjou, B. et al. Design and properties of antimicrobial biomaterials surfaces. *Adv. Healthc. Mater.* **12**, 2202073 (2023). 10.1002/adhm.202202073 36254817

[29] Ran, Y. et al. Fiber-optic theranostics (FOT): interstitial fiber-optic needles for cancer sensing and therapy. *Adv. Sci.* **9**, 2200456 (2022). 10.1002/advs.202200456 PMC9130922 35319824

[30] Zhang, Y. K. et al. Rare earth-doped microfiber bragg grating refractive index sensor with self-photothermal manipulation. *IEEE Photonics J.* **13**, 6800309 (2021).

[31] Gourisankar, S. et al. Rewiring cancer drivers to activate apoptosis. *Nature* **620**, 417–425 (2023). 37495688 10.1038/s41586-023-06348-2 PMC10749586

[32] Nelson, B. J. & Pané, S. Delivering drugs with microrobots. *Science* **382**, 1120–1122 (2023). 38060660 10.1126/science.adh3073

[33] Qiu, M. et al. Novel concept of the smart NIR-light–controlled drug release of black phosphorus nanostructure for cancer therapy. *Proc. Natl Acad. Sci. USA* **115**, 501–506 (2018). 29295927 10.1073/pnas.1714421115 PMC5776980

[34] Yang, K. et al. Low temperature photothermal therapy: advances and perspectives. *Coord. Chem. Rev.* **454**, 214330 (2022). 10.1016/j.ccr.2021.214330

[35] Yi, X. L., Duan, Q. Y. & Wu, F. G. Low-temperature photothermal therapy: strategies and applications. *Research* **2021**, 9816594 (2021). 34041494 10.34133/2021/9816594 PMC8125200

[36] Zhao, R. B. et al. A drug-free tumor therapy strategy: cancer-cell-targeting calcification. *Angew. Chem. Int. Ed.* **55**, 5225–5229 (2016). 10.1002/anie.201601364 26990600

[37] Llovet, J. M. et al. Locoregional therapies in the era of molecular and immune treatments for hepatocellular carcinoma. *Nat. Rev. Gastroenterol. Hepatol.* **18**, 293–313 (2021). 33510460 10.1038/s41575-020-00395-0

[38] Gabizon, A. et al. Prolonged circulation time and enhanced accumulation in malignant exudates of doxorubicin encapsulated in polyethylene-glycol coated liposomes. *Cancer Res.* **54**, 987–992 (1994). 8313389

[39] Masciocchi, C. et al. Uterine fibroid therapy using interventional radiology mini-invasive treatments: current perspective. *Med. Oncol.* **34**, 52 (2017). 28236104 10.1007/s12032-017-0906-5

[40] Song, Q. L. et al. An oral drug delivery system with programmed drug release and imaging properties for orthotopic colon cancer therapy. *Nanoscale* **11**, 15958–15970 (2019). 31418432 10.1039/C9NR03802G

[41] Zhu, J. M. et al. Engineered *Lactococcus lactis* secreting Flt3L and OX40 ligand for in situ vaccination-based cancer immunotherapy. *Nat. Commun.* **13**, 7466 (2022). 36463242 10.1038/s41467-022-35130-7 PMC9719518

[42] Cabibbo, G. et al. Multimodal approaches to the treatment of hepatocellular carcinoma. *Nat. Rev. Gastroenterol. Hepatol.* **6**, 159–169 (2009). 10.1038/ncpgasthep1357 19190599

[43] Liu, Y. et al. Near-infrared radiation-assisted drug delivery nanoplatform to realize blood–brain barrier crossing and protection for parkinsonian therapy. *ACS Appl. Mater. Interfaces* **13**, 37746–37760 (2021). 34318658 10.1021/acsami.1c12675

