# Supplementary material for: Fiber-optic drug delivery strategy for synergistic cancer photothermal-chemotherapy
Source: Light Sci Appl. 2024 Sep 3;13:228. doi: 10.1038/s41377-024-01586-z (PMC11372069; doi:10.1038/s41377-024-01586-z)
Supplement: Supplementary file 1 — Supplemental material for Fiber-optic drug delivery strategy for synergistic cancer photothermal-chemotherapy [file 41377_2024_1586_MOESM1_ESM.docx]

Supplementary Information for

Fiber-optic drug delivery strategy for synergistic cancer photothermal-chemotherapy

*Yongkang Zhang, Jie Zheng, Fangzhou Ji, Jie Xiao, Ni Lan, Zhiyuan Xu, Xu Yue, Zesen Li, Chengzhi Li, Donglin Cao, Yifei Wang, Wenbin Zhong, Yang Ran*, and Bai-Ou Guan**

**Supplementary figures:**


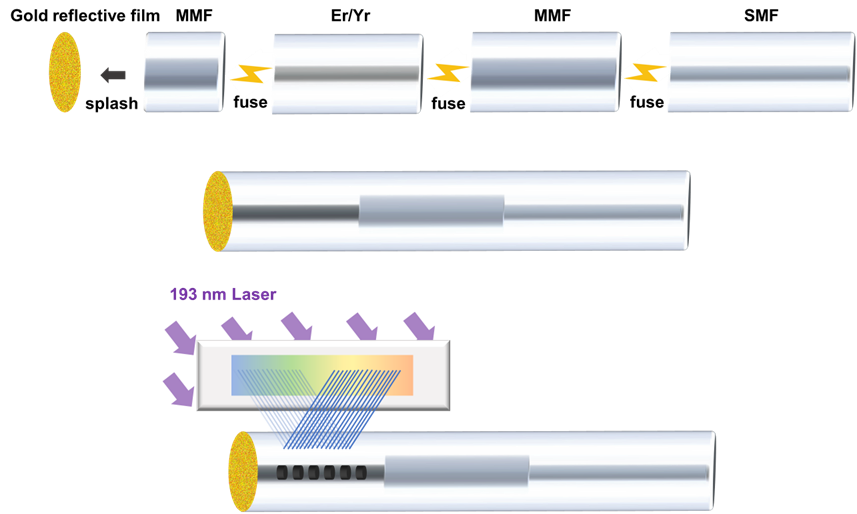


**Figure S1**. The schematic diagram of optic-fiber probe’s fabrication. The MZI used Single mode and Multimode(MSM) structure, and the single-mode fiber was replaced with active fiber. After MZI fabrication, the Bragg grating was inscribed in the active fiber through the phase mask method. A 193 nm UV excimer laser (Compex 110, Coherent, Inc.) and a phase mask with a 1072.5 nm pitch were used for the inscription. The laser had a frequency of 50 Hz and an energy density of 300 mJ cm^-2^ per pulse. A cylindrical lens was used to converge the beam to enhance the energy density. In the process of inscribing, an optical spectrum analyzer (OSA) with a spectrum range of 600-1700 nm and an ultra-wideband light source (Golight) with a range of 1250-1650 nm were needed to demodulate and record the optical signals in this process. The resolution of OSA was set to 0.05 nm to log the tiny spectral variation. The inscription time was about 10 minutes. The gold film was coated on the MZI reflective end by an ion spectrometer (SBC-12, KYKY). Finally, a MZI with FBG was obtained, named “Optic-fiber probe”. MZI: Mach–Zehnder interferometer; FBG: Optical fiber Bragg grating.


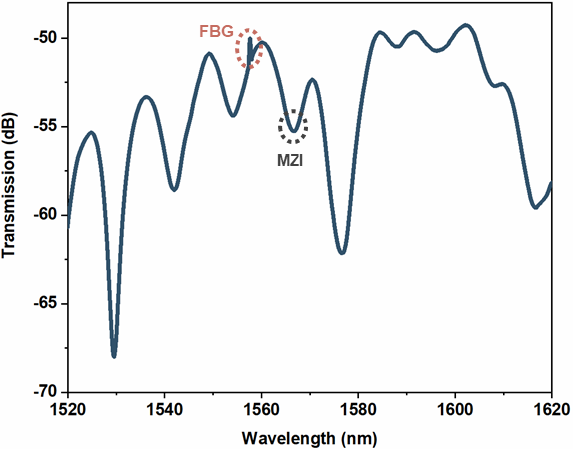


**Figure S2**. The spectrum of optic-fiber probe. The MZI (black) and FBG (red) are combined in only one fiber and the spectrum is overlaid.


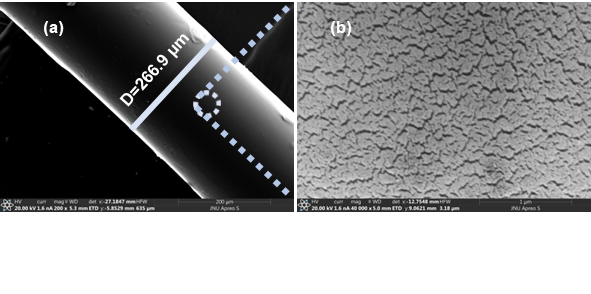


**Figure S3**. SEM image. (a) The SEM image of optic-fiber therapeutic probe loaded with Dox@Agarose. The diameter of probe is about 267 μm, and the thicknesses of Agarose@Dox after drying is 142 μm because the fiber’s diameter is 125 μm. The dried hydrogel demonstrates sufficient adhesion to optical fiber surfaces for *in vivo* experiment. (b) The morphology of agarose on the surface of optic-fiber therapeutic probe after drying.


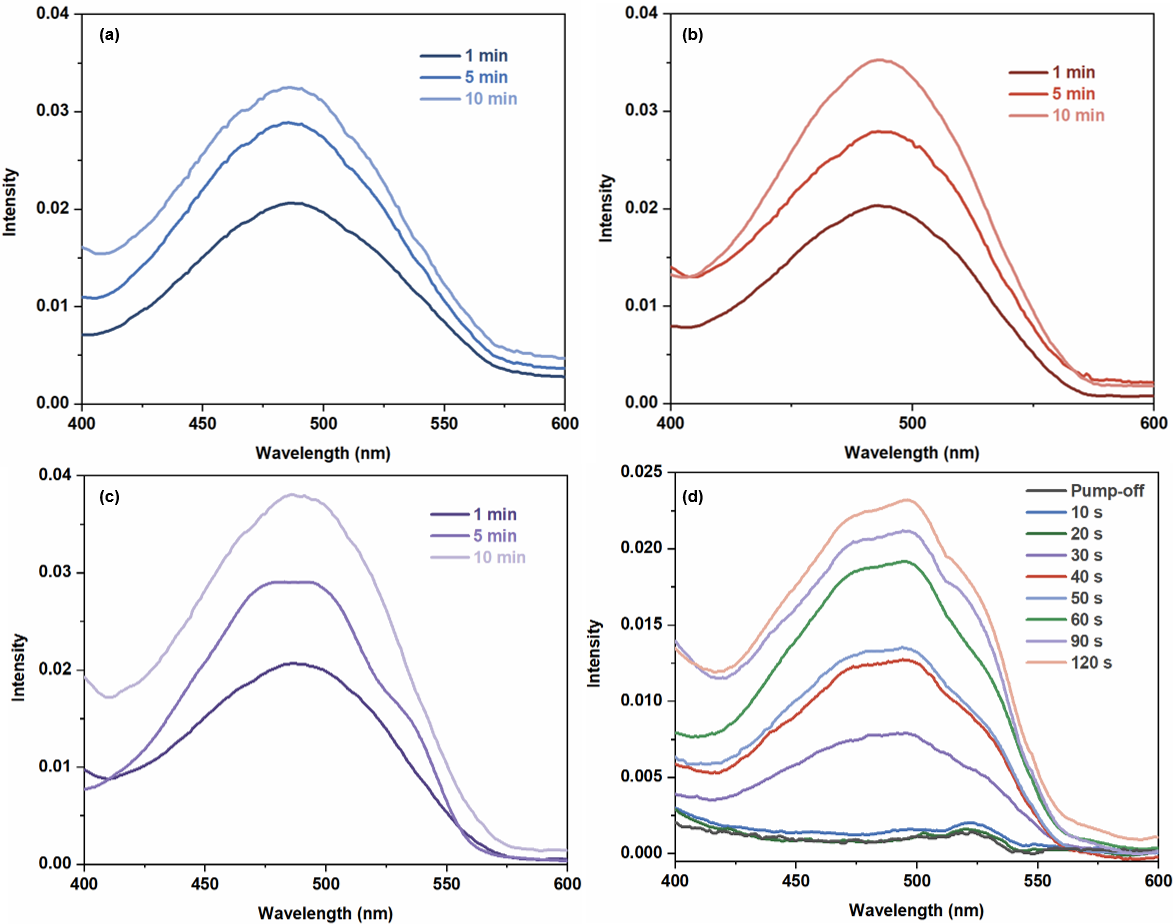


**Figure S4**. The Dox absorption spectra of drug release by optic-fiber therapeutic probe at different times with peak absorption at 500 nm. (a) in first experiment. (b) in second experiment. (c) in third experiment. (d) The absorption spectra of Dox of drug release by optic-fiber therapeutic probe at different times with peak absorption at 500 nm in 2 minutes.


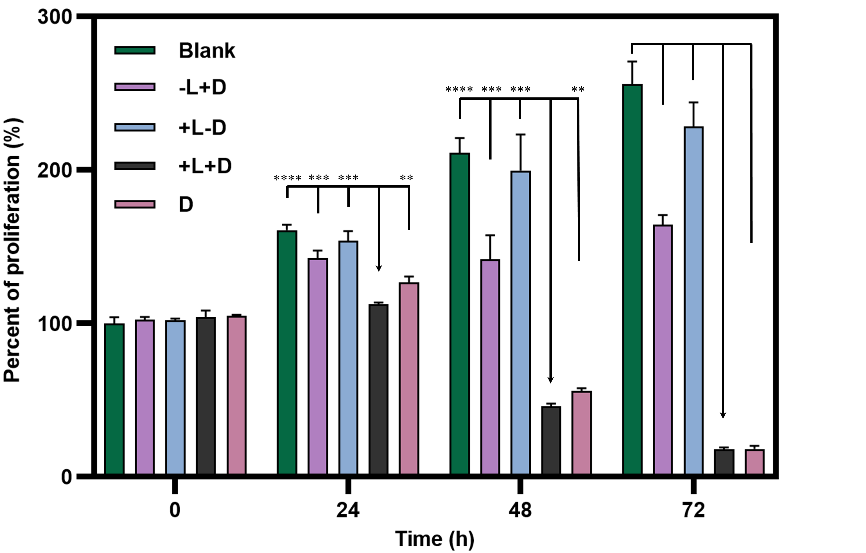


**Figure S5**. Statistical distinctions among the groups at several time *in vitro* cell viability experiment. Statistical analysis is performed by Student's t test (t-test). **p* < 0.05; ***p* < 0.01; ****p* < 0.001; *****p* < 0.001. The +L+D group has significantly statistical distinctions comparing with the Blank control, -L+D, +L-D groups at 24 ,48 and 72 hours, which shows great performance of photothermo-chemotherapy by optic-fiber therapeutic probe *in vitro* cell experiment.


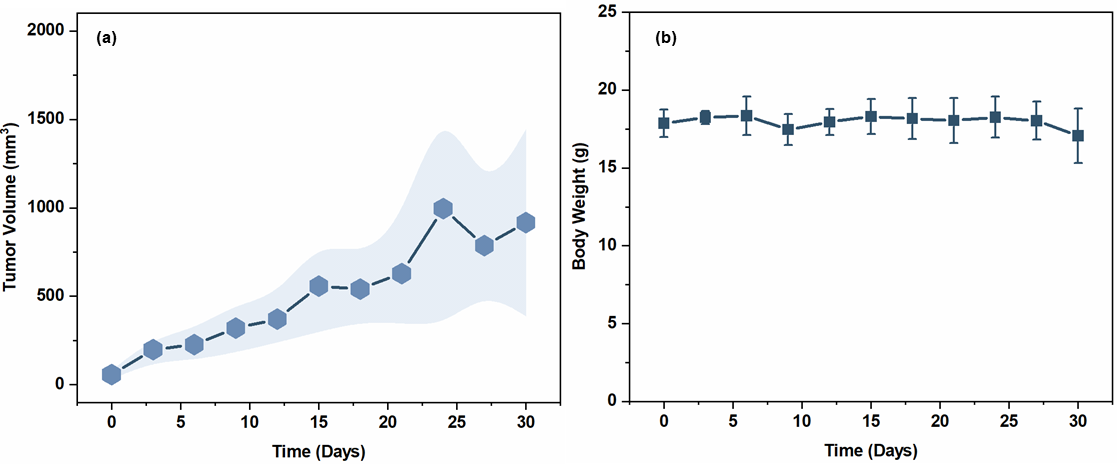


**Figure S6**. The Anticancer efficacy of +Dox groups. (a) The Corresponding growth curves of tumors in +Dox groups (inject the Dox with PBS solution directly) of mice at a measured time; V_0_ means the volume before treatment. The volume (mm^3^) = 1/2 × (tumor length) × (tumor width)^2^ (n=6). (b) Body weight of nude mice recorded every 3 days after treatment.


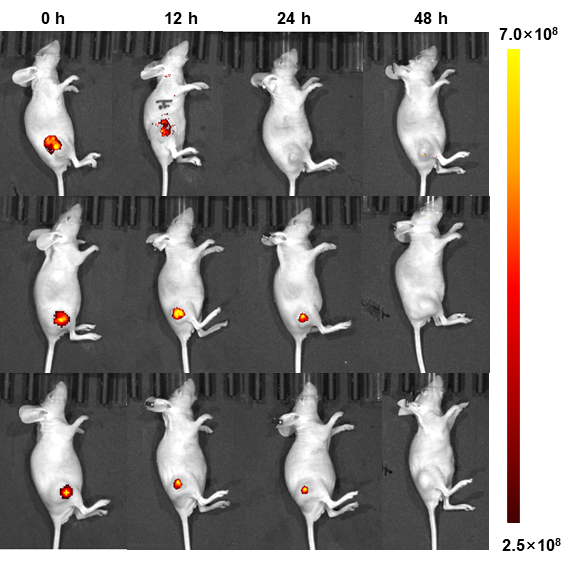


**Figure S7**. The fluorescence images of the +Dox group mice which were injected Dox with PBS solution (Dox concentration was consistent with +D+L group) directly into tumor at 0 h,12 h,24 h, 48 h. The 520 nm wavelength light was used as the excitation source, and 570 nm was detected as the emitted light. The fluorescence lasting for 48 hours is shorten significantly comparing with the +L+D group which shows the TACE-like anticancer effects in the photothermo-chemotherapy based on optic-fiber drug delivery system.


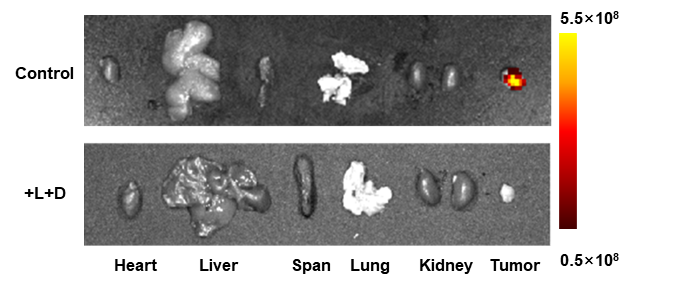


**Figure S8**. The fluorescence images of mice’s organs and tumors 24 hours after the photothermo-chemotherapy. The 520 nm wavelength light was used as the excitation source, and 570 nm was detected as the emitted light. (He, heart; Li, liver; Sp, spleen; Lu, lung; Ki, kidney; Tu, tumor). The fluorescence only appeared at the tumor, proving that drug delivery through optic-fiber therapeutic probe could solve the systemic toxicity of drugs. The fluorescence completely covers the tumor, suggesting that the Dox was diffused over the tumor, which conformed to Figure 6(a).


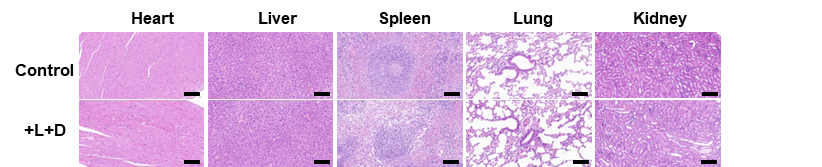


**Figure S9**. The H&E staining of organs after 24 hours in different treatments. The Similar staining results on the organs shows that the drug delivery based on optic-fiber therapeutic probe for cancer could solve the drug toxicity in organs. The scale bars of H&E are 200 μm.


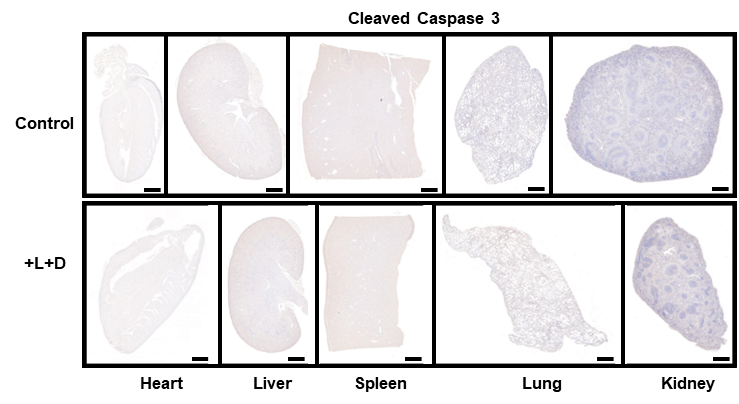


**Figure S10**. The Cleaved Caspase 3 immunohistochemistry of organs. The negligible effects on the organs proves that the drug delivery based on optic-fiber therapeutic probe for cancer could solve the drug toxicity in organs. The scale bars of Cleaved Caspase 3 are 1 mm.


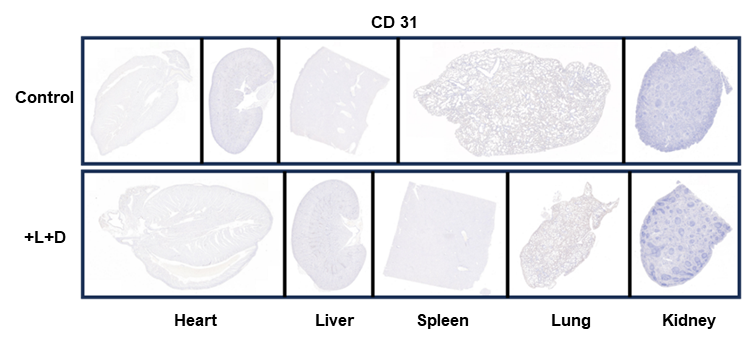


**Figure S11**. The CD31 immunohistochemistry of organs. Nearly identical results on the organs demonstrates that the drug delivery based on optic-fiber therapeutic probe for cancer could solve the drug toxicity in organs. The scale bars of CD 31 are 1 mm.


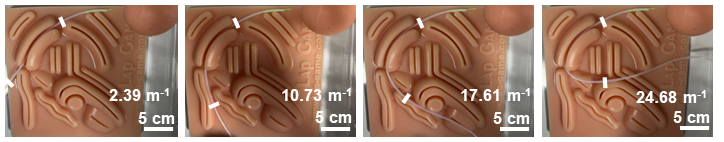


**Figure S12.** The photograph of optic-fiber therapeutic probe combined with interventional therapy at different curvatures. It demonstrates the excellent compatibility of optic-fiber drug delivery system and interventional therapy.

$$\text{Resolution limit}=\frac{3\sigma}{\text{Sensitivity}}$$

**Equation S1.** The formula for calculating the refractive index limit resolution of MZI.

| Group | With 980 pump? | With Dox? |
| --- | --- | --- |
| 1 (Control) | × | × |
| 2 (-L+D) | × | √ |
| 3 (+L-D) | √ | × |
| 4 (+L+D) | √ | √ |
| 5 (D) | × | Inject Dox solution |

**Table S1.** The table with characterizations of different group *in vitro* and *in vivo* experiments.

|  | OP^1^ | TDDS^2^ | TACE^3^ | RADD^4^ | OFDD |
| --- | --- | --- | --- | --- | --- |
| Low systemic toxicity |  |  | √ | √ | √ |
| High delivery efficiency |  | √ | √ | √ | √ |
| Deep therapy | √ | √ | √ |  | √ |
| Long-term retention |  |  | √ | √ | √ |
| Controlled-release |  |  |  | √ | √ |
| Monitorable |  |  |  |  | √ |

**Table S2.** The comparison of different drug delivery strategy. Abbreviations: PO: Per os, oral administration; TDDS: Targeting drug delivery system; TACE: Transcatheter arterial chemoembolization. RADD: Radiation-Assisted Drug Delivery; OFDD: Optic-fiber drug delivery.

**Supplementary method**

*Cell Lines and Cell Culture*

Human hepatocellular carcinoma cell line HepG2 was obtained from the American Type Culture Collection and cultured in Eagle’s Minimum Essential Medium supplied with 10% fetal bovine serum (ExCell Bio, Shanghai, China) and 1% penicillin-streptomycin (Gibco). Cells were cultured in 37 ^o^C with 5% CO_2_.

*CCK-8 assay*

Cell viability was detected by CCK-8 kit (MedChemExpress, American). After treatments, HepG2 cells were seeded into 96-well plates at a density of 1×10^4^ cells well^-1^. Detection was done in 5 wells per group, and blank controls were also detected. At 0, 24 h, 48 h, and 72 h, 10 μL of CCK-8 solution was added, followed by incubation for 3 h at 37°C. The optical density (OD) was measured at 450 nm to reflect the cell viability.

*AO/PI staining*

The AO/PI staining was used to examine morphological changes in HepG2 cells after 3,4-diarylpyrazoles treatment. The HepG2 cells (1×10^6^) were treated with different for 1 min in the ultra-clean bench, and then washed in PBS. Cells were further incubated with 100 μL of AO/PI solution (1 part of 100 μg mL^-1^ of AO in PBS; 1 part of 100 μg mL^-1^ of PI in PBS). After 30 min incubation, cells were washed with PBS and morphology examined under a fluorescence microscope (EVOS FL, Life technologies). Viable cells exhibit intact membrane and produce green fluorescence while apoptotic cells show bright orange color which indicates dead cells. The photographs were captured using inverted fluorescence microscope (EVOS FL, Life technologies at 20x magnification).

*Animals*

The four to five-weeks-old female BALB/c-Nu mice [BALB/cJGpt-Foxn1nu/Gpt] were purchased from Charles River (Foshan, China). Animal experiments were approved by Jinan University's Institute of Experimental Animal Ethics Committee (Approval number: IACUC-20230506-05), and all mice were kept in Laboratory Animal Center, Jinan University. Nude mice were maintained under specific pathogen-free (SPF) conditions for one week before the study.

*Histological and Immunohistochemical Analyses*

The paraffin-embedded tumor tissues were sliced at a thickness of 4 µm. Histological and apoptotic cells were evaluated by H&E staining in tumor tissues and organs (heart, liver, spleen, lung, kidney). The tissue sections were deparaffinized in immunohistochemical analysis. Antigen retrieval was performed using ethylene diamine tetra acetic acid (EDTA) antigen retrieval solution (Beyotime). The slides were blocked with 3% bovine serum albumin (BSA) for 1 hour, subsequently, mixed them with anti-CD31 (GB11063-1, 1:200 dilution, Servicebio), anti-Ki67 (Cat No. 27309-1-AP, 1:200 dilution, Proteintech), and anti-Cleaved caspase 3 (GB11532, 1:600 dilution, Servicebio) antibodies overnight at 4 °C. The slides were washed with PBS (GC305010) and incubated with horseradish peroxidase (HRP)-coupled secondary antibodies, including HRP-coupled anti-mouse (Cat. 7076, 1:400, Cell Signaling Technology), antirabbit (GB23303, 1:200, Servicebio) and goat (GB23303, 1:200 dilution, Servicebio). Then, we stained with a diaminobenzidine (DAB) kit, followed by hematoxylin re-staining. The images were captured with PANNORAMIC DESK/MIDI/250/1000 (3DHISTECH (Hungary)) and analyzed with CaseViewer2.4 software (3DHISTECH (Hungary)). The Tunel assay kit (G1501, Servicebio) were prepared to evaluate the apoptotic cells in tumor tissues, and the images were obtained with ortho-fluorescent microscopy (Nikon Eclipse C1) and an imaging system (Nikon DS-U3). All images were analyzed by Aipathwell (an AI program from Servicebio).

**Supplementary videos**

1. The observation of Dox@Agarose melting by photothermal under the microscope.
2. The observation of drug release by optic-fiber therapeutic probe under the endoscopy.
3. The treatment process of photothermo-chemotherapy based on the fiber-optic drug delivery system.
4. The animation of drug release.
5. The combination of optic-fiber therapeutic probe and interventional therapy.

**References**

1. Song, Q., Jia, J., Niu, X., Zheng, C., Zhao, H., Sun, L., Zhang, H., Wang, L., Zhang, Z., and Zhang, Y. (2019). An oral drug delivery system with programmed drug release and imaging properties for orthotopic colon cancer therapy. Nanoscale *11*, 15958-15970. 10.1039/C9NR03802G.

2. Zhu, J., Ke, Y., Liu, Q., Yang, J., Liu, F., Xu, R., Zhou, H., Chen, A., Xiao, J., Meng, F., et al. (2022). Engineered Lactococcus lactis secreting Flt3L and OX40 ligand for in situ vaccination-based cancer immunotherapy. Nature Communications *13*, 7466. 10.1038/s41467-022-35130-7.

3. Cabibbo, G., Latteri, F., Antonucci, M., and Craxì, A. (2009). Multimodal approaches to the treatment of hepatocellular carcinoma. Nature Reviews Gastroenterology & Hepatology *6*, 159-169. 10.1038/ncpgasthep1357.

4. Liu, Y., Hong, H., Xue, J., Luo, J., Liu, Q., Chen, X., Pan, Y., Zhou, J., Liu, Z., and Chen, T. (2021). Near-Infrared Radiation-Assisted Drug Delivery Nanoplatform to Realize Blood–Brain Barrier Crossing and Protection for Parkinsonian Therapy. ACS Applied Materials & Interfaces *13*, 37746-37760. 10.1021/acsami.1c12675.
